# The Pattern of Pre-hospital Medical Service Delivery in Iran; a Cross Sectional Study

**Published:** 2017-02-19

**Authors:** Mashyaneh Haddadi, Mohammad Sarvar, Hamid Soori, Elaheh Ainy

**Affiliations:** 1 Disaster and Emergency Management Center, Ministry of Health and Medical Education, Tehran, Iran.; 2 Safety Promotion and Injury Prevention Research Center of Shahid Beheshti University of Medical Sciences, Research affair Department, Tehran, Iran.

**Keywords:** Equipment safety, emergency medical service, response latency, ambulances

## Abstract

**Introduction:**

Pre-hospital emergency systems provide service by Franco-German and Anglo American models. This study was carried out to compare the Iranian emergency medical service (EMS) with the two models regarding timing and equipment.

**Methods:**

In this cross sectional study, response time, scene time, and transport time to hospital as well as ambulance equipment of five hundred thousand Tehran EMS recorded missions, during one year, were compared with Franco-German and Anglo American models, trying to determine the pattern of EMS delivery in Iran.

**Results:**

The mean response time, scene time, and transport time to hospital were 15.00 ±10.88, 18 ±11.48, and 15.00 ±11.20 minutes, respectively. The mean response time (p<0.035), scene time (p<0.033), and transport time to hospital (p<0.015) were more than the standard time. Percentage of ambulances quipped with automated external defibrillator (45%, p<0.001), ventilator (2%, p<0.001), disposable splint (0%, p<0.001), and wheelchair (0%, p<0.001) were very far from standards.

**Conclusion:**

The pattern of EMS delivery in Iran was a combination of Anglo American and Franco-German system.

## Introduction

Pre-hospital care with emergency medical services (EMS) can play a major role in the secondary and tertiary prevention (1). Legal Medicine Organization of Iran announced that from 2006 to 2010 more than half of those killed by traffic accidents died at the scene, and 9 to %16 died during transport to the hospital (2). Pre-hospital accident cares initiated by the first proceeding at the scene (informing efficient and rescue forces, rapid delivery of EMS personnel, suitable release, the correct diagnosis, and early treatment measures) and continued during transport to hospital (3). In countries and regions where pre-hospital care is well-designed and good quality services is delivered to victims of traffic accidents, deaths and disabilities from road traffic injuries is greatly reduced. 

The United Nations Road Safety Collaboration has developed a Global Plan for the Decade of Action for Road Safety 2011-2020 with five categories or "pillars" of activities. Improving post-crash response is one of the pillars (4). EMS provides service by two models, namely Franco-German and Anglo American. In Franco-German model, physicians and medical staff can treat the victims with high-tech equipment on the scene and in Germany, France, Greece, Malta, Austria has developed favorably. However, in Anglo American model, which is used in some countries such as United States of America, United Kingdom, Canada, New Zealand, the Sultanate of Oman, and Australia, patients are sent to the hospital quickly with minimal pre-hospital interventions (1, 5). Each of the Franco-German and Anglo American models follow a special pattern and have special education facilities and the provision of services. In some countries, including the UK pre-hospital medical services are offered through the health system (6). In Iran, Disaster and Emergency Management Center of Ministry of Health is responsible for EMS, but the pattern of pre-hospital medical services delivery is not well defined. This study was carried out, to compare the Iranian emergency medical services (EMS) with the two mentioned models regarding response time, scene time, and transport time to hospital.

## Methods

***Study design and setting***

In this cross sectional study, trying to determine the pattern of pre-hospital medical delivery in Iran, response time, scene time, and transport time to hospital of five hundred thousand Tehran EMS recorded missions, over a period of one year (first of August 2015 to first of August 2016), were compared with selected countries: Germany and France (with Franco-German system) and United States and United Kingdom (With Anglo-American system). The protocol of the study was approved by Ethics Committee of Shahid Beheshti University of Medical Sciences. 

***Participants***

Five hundred thousand recorded missions registered by the automation system of Tehran EMS center during the study period were enrolled. Those who attended the emergency centers themselves were excluded because their response time was zero. 

***Data gathering***

The data regarding EMS’s response time, scene time and transport time to hospital were gathered. Data were extracted from global positioning system (GPS) monitored missions registered by the automation system of Tehran EMS center. 

According to the national mandatory standard N0:4374 all ambulances must be equipped. Ambulances’ equipment have been delivered based on the mentioned standard (7).

The data of two studied models regarding the times and needed equipment in pre-hospital care were extracted via a literature search in several databases including PubMed and Transport Research International Documentation (TRID), Cochrane and web of science. The notions of response time, scene time and transport time to hospital were also reviewed in a broad context.

***Definitions***

Most researchers in the recent years have divided the total out of hospital time to a number of “intervals,” including a response time (time from EMS system activation to presence of an ambulance on the scene), scene (or on-scene) time (time that an ambulance spends on the scene to the beginning of departure), and transport time (time from the beginning of departure to patient’s arrival at the emergency department).

***- Franco-German model of pre-hospital care***

In this model: doctor is brought to the patient; care is often provided by emergency physicians; based on the “stay and stabilize” philosophy; fewer transports to the hospital; direct transport to inpatient wards; utilizes more extensive advanced technology; widely implemented in Europe; EMS as part of public health organization (1, 8).

***- Anglo-American model of pre-hospital care***

In this model: patient is brought to the doctor; care provided by emergency medical technicians/paramedics; based upon the “scoop and run” philosophy; more patients transported to the hospital; brought to the emergency department; more likely to be found in countries with emergency medicine as a developed specialty; widely implemented in English speaking countries globally (1, 8).

***Statistical analysis***

All analyses were carried out using Epi Info software (Version 7.2. developed by Centers for Disease Control and Prevention (CDC) in Atlanta, Georgia, USA). Data were reported as mean ± standard deviation or frequency and percentage. A bivariate analysis was conducted using chi square and t test. P values < 0.05 were considered statistically significant.

## Results

***Baseline characteristics***

500000 Tehran EMS recorded missions were studied. Doctors and nurses are present in 4.9% of ambulances (type C ambulances) and in the majority of ambulances (type B ambulances) two technicians are present (95.1%). The education levels of the EMS staff were technicians in 80.4% (60.4% associate degree and 20% with medical emergency training) and bachelors and Master of Science in 19.6% of cases. 

***Comparisons***

Table 1 and 2 compare the studied pre-hospital medical service deliveries with Anglo-American and Franco-German models regarding the times and equipment. The mean response time (p<0.035), scene time (p<0.033), and transport time to hospital (p<0.015) were very far from standards. In addition, percentage of ambulances equipped with automated external defibrillator (45%, p<0.001), ventilator (2%, p<0.001), disposable splint (0%, p<0.001), and wheelchair (0%, p<0.001) was significantly lower than the standards. 100% of studied ambulances were equipped with long backboard, traction splint, and electric suction, similar to Franco-German and Anglo American models. 

**Table 1:** Comparison of studied pre-hospital medical service deliveries with Anglo-American and Franco-German models regarding time management

**Times**	**Iran**	**Anglo-American**	**Franco-German**	**Standard**
**Response **	15.0 (14.6)	10.0 (8.0)	20.9 (15.0)	6.0-8.0
**Scene **	18.0 (17.5)	21.2 (19.0)	32.6 (30.0)	8.0-10.0
**Transport **	15.0 (14.5)	23.3 (20.0)	19.9 (15.0)	15.0-20.0

All times in minutes. Data were presented as mean (median) or range.

**Table 2:** Comparison of studied pre-hospital medical service deliveries with Anglo-American and Franco-German models regarding equipment

**Equipment**	**Iran**	**Anglo-American**	**Franco-German**	**Standard**
**AED** *	45	60	60	100
**Ventilator**	2	100	100	100
**Long backboard**	100	100	100	100
**Traction splint**	100	100	100	100
**Disposable Splint**	0	30	50	100
**Wheelchair**	100	100	100	100
**Vacuum suction**	100	100	100	100

* : Automated external defibrillator. Data were presented as percentage and extracted from following references (1, 5, 6, 8, 10-13, 23-26).

## Discussion

Findings showed that the pattern of EMS delivery in Iran was a combination of Anglo American and Franco-German system. Pre-hospital emergency medical service of Iran was reachable through a nationwide 115 number, but other numbers such as, 110 (Police), 112 (Rescue), and 125 (fire) to report traffic accidents and relief services can also be used. Notification to relief centers is conducted with delay due to lack of Universal Access Number (UAN) and the Joint Dispatch. Creating a unique relief number reduces the rate of delay in relief. 

It seems that Franco-German emergency system has significant drawbacks compared to Anglo American system. In Anglo American model the educational level of EMS staff is as follows: 13.2% physicians, 84.5% nurses, and 2.3% emergency medical technicians (9). In Franco-German system physicians and medical staff should treat the victims with high-tech equipment on the scene. Emergency trauma patients are treated at the scene of an accident and during transport by emergency physicians. Rescuers are often the first to arrive at the accident scene and may use defibrillator, carry out endotracheal intubation, and administer some drugs such as endotracheal epinephrine and intravenous glucose until reaching the doctor. It is guaranteed that the emergency patients are reached in up to 8 minutes in %80 and 15 minutes in %95 of the cases (10-16). In Franco-German model qualified emergency physicians and paramedics provided standard care. The entire pre-hospital time and scene time is just slightly longer than Anglo American system. There are specific training programs for emergency physicians, EMS staff and medical director of Emergency system managers. Resuscitation is not only performed by anesthesiologists but also internists, surgeons or pediatricians. Medical Emergency includes cardiopulmonary resuscitation, the shock cases, myocardial infarction, acute stroke, poly-trauma, and status asthma. In Franco German system incidence of mortality in trauma victims has decreased from 830 thousand annual deaths (%10) to less than %4 (17, 18). The Anglo-American model is bringing patients to the hospital with less pre-hospital interventions quickly. The system has been run by trained paramedics and Emergency Medical Technicians with a clinical supervision. Countries which use Anglo-American model of EMS delivery include the United States, United Kingdom, Canada, New Zealand, Sultanate of Oman and Australia (19-22). It seems that EMS must be presented by an organization that is capable of delivering the best health care services and works with other healthcare providers as well as social service and public safety agencies. The pattern of emergency in Iran was a combination of Anglo American and Franco-German systems. Factors such as traffic, poor roads, and remote and out of reach areas, and inadequate relief helicopters are the causes of delay for pre-hospital care in the emergency cases. Pre-hospital services deviate from standards due to lack of adequate equipment. On the other hand, due to the lack of safety standard in auto industry of Iran, severity of injury is high in car accidents. It is proposed that pre hospital emergency system of Iran should follow a single pattern either Franco German or Anglo American system and have the required equipment according to the selected pattern. Automobile safety must be strongly supervised by authorities in automotive industry. The number of emergency air ambulances must be increased. Urban and rural roads must be modified by the ministry of roads and urban development. According to the condition of cities, equipped emergency bases must be built in high risk areas. Iranian emergency personnel should be supported financially and psychologically for their hard work. 

The study was carried out for the first time in Iran and five hundred thousand missions have been investigated. Study was conducted on registered missions of Tehran emergency over a one year period that could be the strength of the study. 

***Limitations***

This was a cross sectional study. Cross sectional studies provide a snapshot of the frequency of a disease or other health related characteristics in a population at a given point in time. Method of treatment and patients’ satisfaction were not recorded. These could be the limitations of the study. 

## Conclusion:

The pattern of EMS delivery in Iran was a combination of Anglo American and Franco-German system. Therefore, it is suggested to follow a single pattern and be equipped in accordance with the selected model.

## Notes

**Funding: **No funds
